# The Frequency of Extra-Uterine Pregnancy

**Published:** 1898-03-12

**Authors:** 


### THE FREQUENCY OF EXTRA-UTERINE PREGNANCY.

In an article on extra-uterine pregnancy which appeared lately in the *New York Medical News* a remark is made in regard to the frequency of that abnormality, to which the attention of general practitioners may well be directed, for we fear it is still too much the habit with many to regard extra-uterine pregnancy as a thing quite out of the common way. "Some idea of the comparative frequency of ectopic pregnancy may be gathered," it is said, "from the fact that, according to the records of the Paterson General Hospital (which, as its name implies, is a general hospital, and not a special institution for women), seven women have been operated upon for ruptured tubal pregnancy during the past eight months. These were all cases of rupture into the peritoneal cavity." Now Paterson is not a very large place. In 1890 its population was 78,347.

